# Metal-N_4_@Graphene as Multifunctional Anchoring Materials for Na-S Batteries: First-Principles Study

**DOI:** 10.3390/nano11051197

**Published:** 2021-05-01

**Authors:** Kaishuai Yang, Dayong Liu, Yiling Sun, Zhengfang Qian, Shengkui Zhong, Renheng Wang

**Affiliations:** 1 Key Laboratory of Optoelectronic Devices and Systems of Ministry of Education and Guangdong Province, College of Physics and Optoelectronic Engineering, Shenzhen University, Shenzhen 518060, China; ksyang@szu.edu.cn (K.Y.); zq001@szu.edu.cn (Z.Q.); 2 Key Laboratory of Materials Physics, Institute of Solid State Physics, HFIPS, Chinese Academy of Sciences, Hefei 230031, China; dyliu@theory.issp.ac.cn; 3 School of Marine Science and Technology, Hainan Tropical Ocean University, Sanya 572000, China; zskui74@163.com

**Keywords:** anchoring materials, shuttling effect, Na-S battery, metal-N_4_@Graphene, first-principles theory

## Abstract

Developing highly efficient anchoring materials to suppress sodium polysulfides (NaPSs) shuttling is vital for the practical applications of sodium sulfur (Na-S) batteries. Herein, we systematically investigated pristine graphene and metal-N_4_@graphene (metal = Fe, Co, and Mn) as host materials for sulfur cathode to adsorb NaPSs via first-principles theory calculations. The computing results reveal that Fe-N_4_@graphene is a fairly promising anchoring material, in which the formed chemical bonds of Fe-S and N-Na ensure the stable adsorption of NaPSs. Furthermore, the doped transition metal iron could not only dramatically enhance the electronic conductivity and the adsorption strength of soluble NaPSs, but also significantly lower the decomposition energies of Na_2_S and Na_2_S_2_ on the surface of Fe-N_4_@graphene, which could effectively promote the full discharge of Na-S batteries. Our research provides a deep insight into the mechanism of anchoring and electrocatalytic effect of Fe-N_4_@graphene in sulfur cathode, which would be beneficial for the development of high-performance Na-S batteries.

## 1. Introduction

In recent years, it has become more and more difficult for the current commercial lithium-ion batteries (LIB), due to their relatively low theoretical energy density limits (200–300 Wh/kg), to meet the ever-growing demands of society’s electrical energy storage, including portable electronic devices, electric vehicles, and smart grid storage applications [1,2,3]. Therefore, novel rechargeable batteries with a large charge storage capacity and a high energy density are urgently needed [4,5,6,7,8,9]. Owing to the extremely high theoretical specific capacity of the elements sulfur (1672 mAh/g) and sodium (1165 mAh/g), and to the batteries’ high theoretical energy density of 1274 Wh/kg of cell weight [10,11,12,13], the rapidly developing room temperature (RT) sodium-sulfur (Na-S) batteries are widely considered as one of the most promising alternative candidates for the next-generation rechargeable batteries to replace the conventional insertion-type LIB [14,15,16,17]. Furthermore, both sodium and sulfur element materials are earth-crust abundant, sufficiently low-cost, and environmentally friendly [18,19].

Despite these advantages, RT Na-S batteries have not yet been practically applied, since this technology still suffers from several critical challenges [17,20,21,22], similar to lithium sulfur (Li-S) batteries: (1) sulfur and the discharge by-products sodium polysulfides (NaPSs) (Na_2_S_n_, where 1 ≤ n ≤ 8) have poor electrical conductivity, which leads to a low utilization of active materials; (2) the high-order NaPSs (Na_2_S_n_, 4 ≤ n ≤ 8) are prone to dissolve in the ether-based liquid electrolyte, then diffuse towards the sodium anode, thus causing the so-called “shuttling effect” and leading to low coulombic efficiency and rapid capacity fading; (3) the large volume expansion of sulfur cathode during discharge can bring about poor stability of the electrode. To address the above-mentioned issues, designing appropriate multifunctional conductive anchoring materials that can both catch and catalyze the NaPSs is of vital importance.

Graphene nanosheet is one of the widely used electrode host materials due to its high specific surface area, attractive electrical/thermal conductivity, and excellent flexibility property [23,24,25]. However, the physical combination between nonpolar pristine graphene and polar polysulfides is too weak to effectively hinder polysulfides shuttling between anode and cathode during charge/discharge cycling. One effective strategy to improve the interactions with polysulfides is to dope heteroatom into graphene nanosheet, based on the lessons learned from Li-S batteries [26,27,28]. Particularly, N atom is the most widely used dopant for Li/Na-S batteries as an anchoring material [26,27,29,30]. For instance, pyrrolic and pyridinic N-doped graphene shows a much stronger combination with lithium polysulfides (LiPSs) through covalent bonds than pristine graphene, as reported by J. J. Chen et al. [26] and Y. Qiu et al. [27]. M. Sajjad et al. reported that polar nitrogenated holey graphene shows a superior anchoring of NaPSs [29]. Additionally, the co-doped graphene/carbon nanostructures are also reported to be intensively attractive, due to their novel geometries and properties [31,32,33,34,35]. For example, G. Xia et al. reported that N and O co-doped porous carbon nanofibers could effectively alleviate the “shuttling effect” via adsorbing NaPSs by strong chemical interactions [31]. J. Yang et al. reported that the N and S co-doped porous carbon nanosheets increase the utilization of sulfur and display excellent rate performance of Na-S batteries [32]. Interestingly, transition metal elements doping is advantageous over non-metallic doping in Li/Na-S batteries, benefiting from the electrocatalytic property of transition metal originated from its special electronic orbital arrangement [28,36,37,38,39]. For instance, B. W. Zhang et al. reported that transition metal (such as Co, Fe, Cu, and Ni) nanoclusters that were decorated in hollow carbon nanospheres could significantly polarize sulfur host to improve the reactivity of S and inhibit the “shuttling effect”, and their electrocatalytic effects are also clearly evidenced by experiments and calculations [37,38]. In particular, several recent experiments and simulations reported that transition metal and nitrogen co-doped graphene exhibit superior performance with good electrocatalysis and shuttling suppression abilities [28,33,40,41,42]. For examples, Q. Jia et al. revealed the high catalytic property of Fe-N co-doped graphene fragments through experimental observations [33]. W. Lai et al. experimentally showed that cathodes with Fe-N co-doped carbon fibers exhibit outstanding rate capacity and cycling performance in RT Na-S batteries [42]. Zhang and co-workers reported that Fe-N_4_ and Cr-N_4_ co-doped graphene shows a strong adsorption and full discharge of LiPSs, resulting in a greatly improved performance of Li-S batteries [41]. However, transition metal and nitrogen co-doped graphene (metal-N_4_@graphene) used in Na-S batteries as multifunctional anchoring materials have been less explored up to now.

Inspired by the aforementioned investigations, here, we systemically investigated the experimentally available transition metal-N_4_@graphene (metal = Fe, Co, and Mn) [42] to illustrate its anchoring and electrocatalytic effects in Na-S batteries by first-principles density functional theory (DFT). Our results demonstrate that Fe-N_4_@graphene can tightly combine with NaPSs through chemical bonds, and its electric conductivity is well retained. Hence, properly doping Fe-N_4_ into graphene can greatly enhance the anchoring effect for NaPSs to suppress the adverse “shuttling effect” in Na-S batteries. Additionally, the doping transition metal ions can effectively lower the decomposition energy barriers of NaPSs, resulting in a signal development of their full discharge. Our results not only facilitate a deep understanding of the mechanism of metal-N_4_@graphene used as a multifunctional anchoring material, but also provide worthy guidance to exploit high-performance Na-S batteries.

## 2. Computational Method

The calculations of this work have been performed within the framework of the DFT by using Vienna ab initio simulation package (VASP) [43,44] code. The Project-Augmented-Wave (PAW) [45] and the Perdew-Burke-Ernzerhof (PBE) Generalized Gradient Approximation (GGA) [46] that are implemented in VASP are carried out for pseudopotentials and the exchange-correlation functionals. Spin polarized calculations are employed for all the co-doped systems, due to the magnetic nature of the transition metal atoms (metal = Fe, Co, and Mn). A plane wave cutoff of 520 eV is used for the kinetic energy of all the graphene and metal-N_4_ doped graphene monolayers. A 3 × 3 × 1 Monkhorst-Pack [47] k-mesh is used for the Brillouin zone integrations of all supercells in all the calculations. About 20 Å vacuum space along the direction perpendicular to the plane of the monolayer is inserted to eliminate the interactions of the adjacent periodic images. All of the structural optimizations are performed with an energy convergence criterion of 10^−5^ eV/cell. The van der Waals (vdW) interactions are described by using the Lee et al. vdW-DF2 functionals [48,49], which have been widely and successfully used to describe co-doped graphene systems [28,40]. To estimate the energy barriers of Na ion diffusion on the surface of pristine graphene and Fe-N_4_@Graphene monolayers, we constructed 2, 4, 4, and 3 linear-interpolation intermediate images between the initial and final Na positions along the pathway of Na_2_S/Graphene, Na_2_S_2_/Graphene, Na_2_S/Fe-N_4_@Graphene, and Na_2_S_2_/Fe-N_4_@Graphene to perform the climbing-image nudged elastic band (ci-NEB) algorithm [50,51], respectively. In ci-NEB calculations, supercells with a single Na adatom are performed, and all of the image configurations are relaxed until the maximum force acting on every atom is less than 0.03 eV/Å. In ci-NEB calculations, we removed one of the sodium ions of Na_2_S and Na_2_S_2_ on graphene and Fe-N_4_@graphene from their most stable adsorption sites, and placed them far away, then optimized their structural configurations and used them as the final states of ci-NEB, respectively. The Fermi energy level of projected density of states (PDOS) has been converted to zero.

The adsorption energies *E*_ad_ of NaPSs molecule on pristine graphene and metal-N_4_@graphene are defined as the following formula:

*E*_ad_ = *E*_sub_ + *E*_mole_ − *E*_tot_ (1)

where *E*_tot_ is the total energy of the substrate with adsorbed NaPSs, *E*_sub_ is the energy of substrate of pristine graphene or metal-N_4_@graphene, and *E*_mole_ is the energy of isolated NaPSs molecule. According to this definition, a positive value indicates an exothermic (energetically favorable) reaction, and the strength of absorbing capability of substrate for NaPSs molecules enhances as the value becomes more positive.

## 3. Results and Discussion

### 3.1. Structures and Electronic Properties of Na_2_S_n_ Species and Metal-N_4_@Graphene

**Figure 1:**
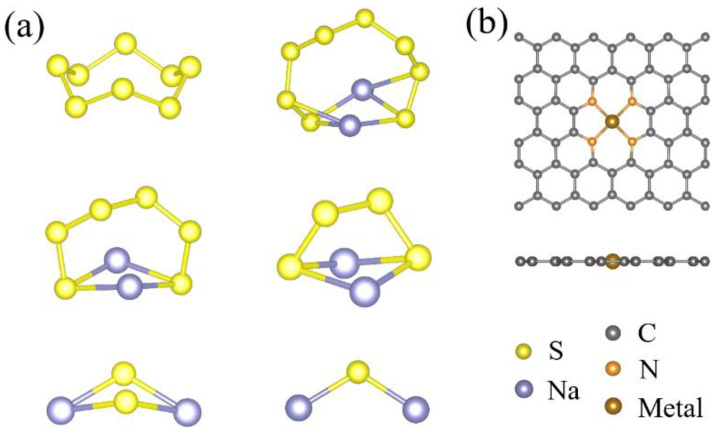
(**a**) The optimized atomic configurations of (**a**) isolated cyclo-S_8_ and Na_2_S_n_ (n = 8, 6, 4, 2, and 1) species. (**b**) The top and side views of optimized metal-N_4_@graphene substrate.

During the discharge process of Na-S batteries, the sodium polysulfide species (typically including Na_2_S, Na_2_S_2_, Na_2_S_4_, Na_2_S_6_, and Na_2_S_8_) are formed, and the final product is Na_2_S species, as demonstrated by experiments [16,52]. The optimized geometries of Na_2_S_n_ (n = 1, 2, 4, 6, and 8) species and cyclo-S_8_ are shown in Figure 1a, and all of these S-containing molecules are in a three-dimensional shape, which are well consistent with previous studies [29,52], suggesting the reliability of our employed calculations. The specific structural parameters of Na_2_S_n_ species and cyclo-S_8_ are listed in Table 1. As the structural data shows, the Na-S bond lengths (~2.50 Å) of low-order Na_2_S_n_ (n = 1, 2) species are significantly shorter than those (~2.75 Å) of high-order Na_2_S_n_ (n = 4, 6, 8) species clusters, while the S-S bonds slightly decrease with the increase in the number of S atoms, similar to the scenario of Li_2_S_n_ species [28,36]. Generally, a longer bond length indicates a weaker chemical binding for the same kind of bond. As a result, the high-order Na_2_S_n_ species are more easily dissolved into sodium cations and polysulfide anions than low-order Na_2_S_n_ species in the electrolyte of Na-S batteries.

Figure 1b presents the optimized structure of transition metal and nitrogen co-doped graphene (metal-N_4_@graphene) substrate in a 3 × 5 supercell (containing 59 atoms), in which all of the atoms are in the same plane. For the metal-N_4_@graphene, six carbon atoms in the center of the substrate are removed and then replaced by four nitrogen atoms and one metal atom. Three kinds of transition metal (Fe, Co, and Mn) atoms are considered in this work. Our calculations show that the central transition metal atom is surrounded by four equivalent metal-N bonds, with the lengths ranging from 1.89 Å to 1.96 Å, which is well consistent with previous literature [28,36].

**Figure 2:**
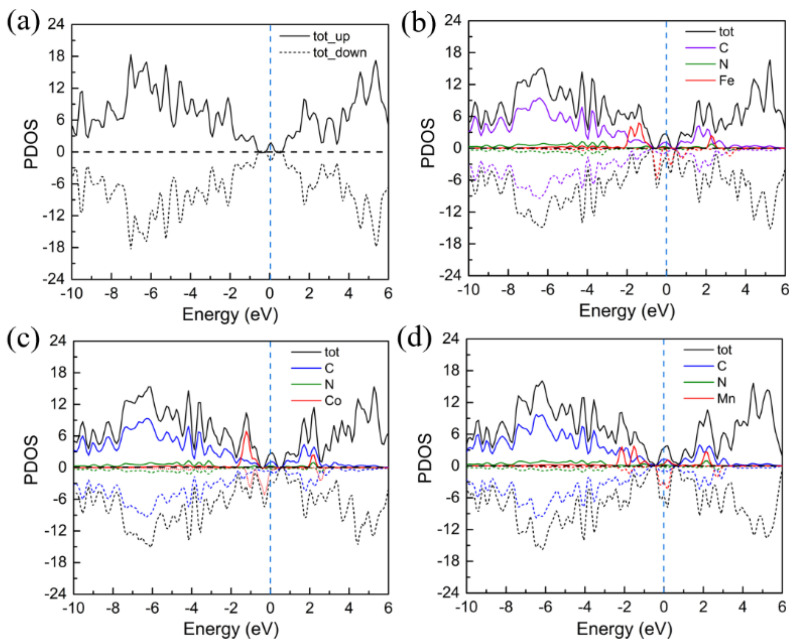
The projected density of states (PDOS) of pristine graphene (**a**) and metal-N_4_@graphene (metal = Fe (**b**), Co (**c**), and Mn (**d**)). The solid and dashed lines represent spin-up and spin-down components of PDOS, respectively. The vertical blue dashed lines represent the Fermi energy level.

In addition, the transition metal-N_4_ co-doped graphene heterostructure enhances its electrical conductivity. Previous studies found that an electrode structure composed with graphene can facilitate electron and ion transport [23,24]. Moreover, heterostructures provide an optimal way for tuning the electronic properties [36]. Based on these reports, the electronic conductivities of co-doped graphene monolayers are investigated; thus, the projected density of states (PDOS) of pristine and transition metal-N_4_ (metal = Fe, Co, and Mn) co-doped graphene are calculated, as illustrated in Figure 2. As shown in Figure 2b–d, the PDOSs of transition metal-N_4_@graphene exhibit spin asymmetrically polarized ground states, due to the difference between the number of electrons in spin-up and spin-down components. In addition, their magnetic moments are mainly localized on the central metal atom, as shown in Table S1. Furthermore, the magnetism of doped iron is mainly contributed from its relatively localized 3*d* orbital electrons (see Figure S4). Notably, there are more PDOSs of transition metal-N_4_@graphene near the Fermi energy than of pristine graphene (see Figure 2). Therefore, the electrical conductivity of metal-N_4_@graphene is effectively improved, which is beneficial for the performance of the cathode electrode.

### 3.2. Adsorption of Na_2_S_n_ Species on Pristine and Transition Metal-N_4_ Co-Doped Graphene

**Figure 3:**
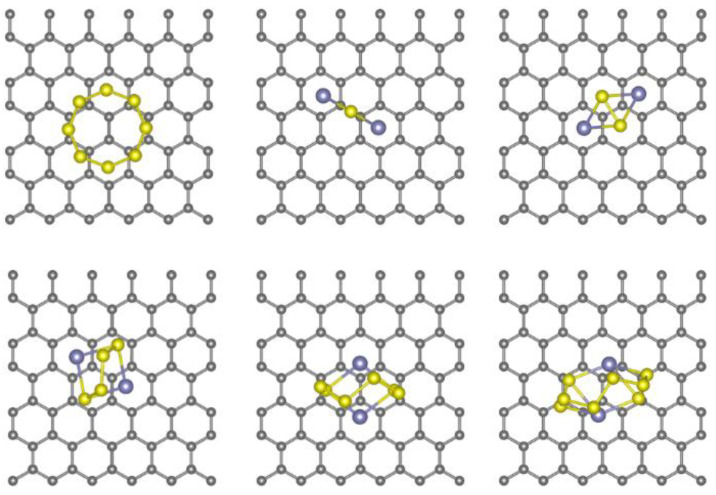
The optimized structures of cyclo-S_8_ and Na_2_S_n_ (n = 1, 2, 4, 6, and 8) species adsorbed on the surface of pristine graphene. The black, blue, and yellow balls represent carbon, sodium, and sulfur atoms, respectively.

To evaluate the potentials of transition metal-N_4_@graphene (metal = Fe, Co, and Mn) as the anchoring material for high-performance Na-S batteries, the adsorption energies of Na_2_S_n_ species on pristine and co-doped graphene are calculated, respectively. The computing results show that the minimum distance between Na_2_S_n_ species and pristine graphene substrate is larger than 2.8 Å, and all adsorption energies of Na_2_S_n_ species on graphene are around 0.7 eV (see Table S2), indicating that there are no chemical bonds between Na_2_S_n_ species and graphene (see Figure 3). The weak van der Waals interactions are not strong enough to stabilize the adsorption of Na_2_S_n_ species; hence, pristine graphene is not a proper anchoring material, which is consistent with previous reports [24,53]. As a consequence, the co-doped graphene as absorbing Na_2_S_n_ species materials are studied by DFT calculations.

**Figure 4:**
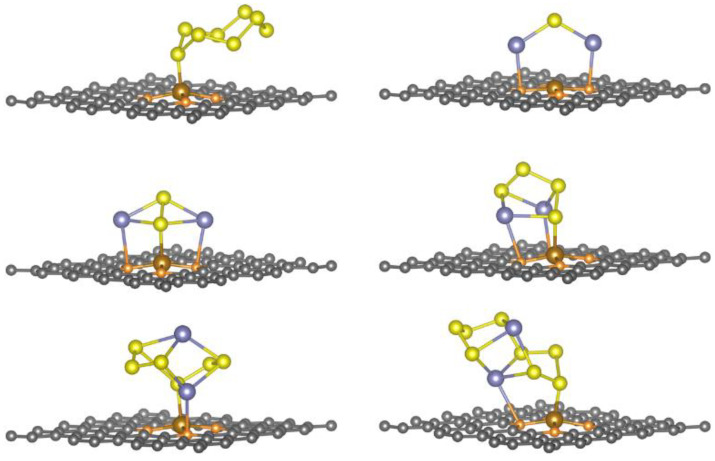
The optimized structures of cyclo-S_8_ and Na_2_S_n_ (n = 1, 2, 4, 6, and 8) adsorbed on the surface of Fe-N_4_@graphene. The black, blue, yellow, orange, and brown balls represent carbon, sodium, sulfur, nitrogen, and iron atoms, respectively.

To obtain the most stable adsorption configurations, we considered different orientations of Na_2_S_n_ species and cyclo-S_8_ at different sites of the Fe-N_4_@graphene surface (see Figure S1). Take Figure S1a,b as an example. The cyclo-S_8_ prefers to parallelly adsorb to the surface of Fe-N_4_@graphene with a minimum distance between them of 2.111 Å. Meanwhile, the shape of adsorbed cyclo-S_8_ has a somewhat structural distortion, that is, the S-S bond is slightly stretched from 2.097 Å of the isolated cyclo-S_8_ molecule to 2.175 Å of the counterpart, and the central Fe atom is pulled outward from the surface of doped graphene by about 0.413 Å, suggesting the formation of an obvious chemical S-Fe bond. After full structural optimization, the most stable configurations of Na_2_S_n_ species and cyclo-S_8_ adsorbed on transition metal-N_4_@graphene are presented in Figure 4, and Figures S2 and S3, and the corresponding key binding parameters are summarized in Table 2, and Tables S3 and S4. In addition, comparing before and after adsorption, metal-N_4_@graphene (metal = Fe, Co, and Mn) as anchoring materials can maintain their structures well, and the Na_2_S_n_ species and cyclo-S_8_ adjust themselves to achieve the most stable adsorption.

**Figure 5:**
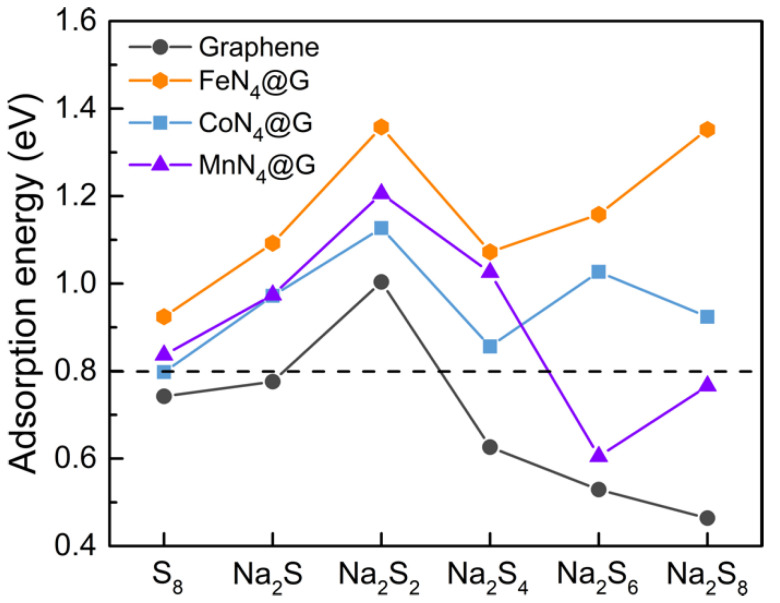
The computed adsorption energy profile of cyclo-S_8_ and Na_2_S_n_ (n = 1, 2, 4, 6, and 8) species after absorbing on pristine and transition metal-N_4_@graphene (metal = Fe, Co, and Mn) surface, respectively. The dashed line represents the critical energy of weak physical adsorption.

As Figure 4, and Figures S2 and S3, show, for metal-N_4_@graphene (metal = Fe, Co, and Mn), one sulfur atom binds with the metal atom, and one or two sodium atoms bind with nitrogen atoms, forming a chemical binding ring configuration. For instance, the chemical ring of the Na_2_S adsorbed system has two Na-N bonds, and the Na_2_S_2_ adsorbed system has two Na-N bonds and one S-metal bond. All adsorption energies of Na_2_S_n_ species on Fe-N_4_@graphene increase significantly and are much greater than that of pristine graphene. Thus, it is obvious that the strong chemical rings with large adsorbing energies make the adsorption configuration more stable, resulting in a high affinity for Na_2_S_n_ species. This feature of co-doped graphene can significantly hinder the shuttling effect, which mainly comes from high-order Na_2_S_n_ species. Notably, the adsorption energies of Na_2_S_n_ species on Fe-N_4_@graphene are obviously larger than that of Co/Mn-N_4_@graphene (see Figure 5), especially for high-order Na_2_S_n_ species, which means that the high-order Na_2_S_n_ species tend to adsorb on Fe-N_4_@graphene much easier than Co/Mn-N_4_@graphene. This indicates that Fe-N_4_@graphene, as the best one among the considered anchoring materials, can most effectively adsorb Na_2_S_n_ molecules to prevent the shuttling effect.

Particularly, after Na_2_S and Na_2_S_2_ adsorbing on Fe-N_4_@graphene, the Na-S bond length prolongs more significantly than in the isolated state (as shown in Table 1 and Table 2). The average bond lengths of Na-S increase from 2.464 Å and 2.581 Å to 2.514 Å and 2.679 Å for Na_2_S and Na_2_S_2_, respectively. The extension of the Na-S bond makes it easier for it to be broken, which is beneficial to the detachment of sodium ion. Therefore, the mechanism of Fe-N_4_@graphene on the decomposition of Na_2_S and Na_2_S_2_ needs to be intensively studied.

### 3.3. Electrocatalytic Performance of Iron for Na_2_S_n_ Species on Fe-N_4_@Graphene

It is worth noting that the decomposition of the deep discharge products (e.g., Na_2_S and Na_2_S_2_) on anchoring materials is a critical factor for energy capability, utilization of sulfur, and cycling performance of Na-S batteries. As the abovementioned analysis shows, Fe-N_4_@graphene is the most effective anchoring material for Na_2_S_n_ species; thus, its electrocatalytic performance deserves further study, which is essential for coulombic efficiency, rate capability, and charge/discharge performance of Na-S batteries. To gain an in-depth understanding of the electrocatalytic property of doped iron, the climbing-image nudged elastic band (ci-NEB) method is applied to calculate the energy barrier for Na_2_S and Na_2_S_2_ decomposing on the surface of pristine graphene and Fe-N_4_@graphene, respectively, to evaluate the sodium extraction reaction kinetics.

**Figure 6:**
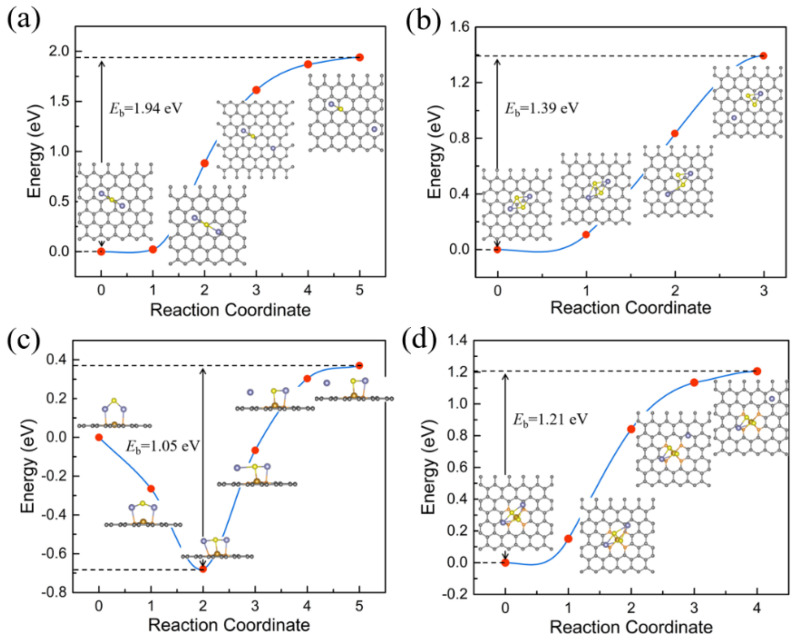
Decomposition pathways and corresponding energy barriers for Na_2_S and Na_2_S_2_ on pristine graphene (**a**,**b**) and Fe-N_4_@graphene (**c**,**d**) surface, respectively. The insertions are the intermediate images of one Na ion departing from the NaS/NaS_2_ cluster.

Herein, we considered the decomposition process from an intact Na_2_S/Na_2_S_2_ molecule into a NaS/NaS_2_ cluster and a single Na ion (Na_2_S/NaS_2_→NaS/NaS_2_ + Na^+^ + e^−^). The main evolution is accompanied by one sodium ion separating and moving away from the sulfur atom in Na_2_S/Na_2_S_2_ molecule, including the breaking of one Na-S bond. The energy profiles for the decomposition processes of sodium ion on pristine graphene and Fe-N_4_@graphene are shown in Figure 6, where the corresponding energy barrier heights are inserted nearby. Our results show that pristine graphene possesses a great decomposition barrier (1.94 eV and 1.39 eV for Na_2_S and Na_2_S_2_, respectively), much larger than that of Fe-N_4_@graphene (1.05 eV and 1.21 eV for Na_2_S and Na_2_S_2_, respectively). It is known that reducing the decomposition barrier of Na_2_S/Na_2_S_2_ can greatly increase the utilization of active materials, decrease the formation of dead polysulfides, and achieve a high charging rate and a longer cycling life. In brief, Fe-N_4_@graphene demonstrates a much lower decomposition barrier of Na_2_S/Na_2_S_2_, which exhibits its promising potential for catalyzing polysulfides in the charging process of Na-S batteries.

In one word, the anchoring material Fe-N_4_@graphene can not only effectively adsorb the sodium polysulfides to block the shuttling effect, but also significantly improve the electronic conductivity and catalyze the electrochemical interactions, consequently achieving multiple targets and addressing several vital challenges. Thus, our research reveals the multi-functional anchoring effects of Fe-N_4_@graphene in Na-S batteries, while providing valuable guidance for designing high-performance sulfur cathode materials.

## 4. Summary

Based on first-principles theory calculations, we systematically investigated the transition metal-N_4_@graphene (metal = Fe, Co, and Mn) as multi-functional anchoring materials to hinder the “shuttling effect” of NaPSs in Na-S batteries. Our calculation results reveal that doping with metal-N_4_@graphene could not only dramatically improve the electronic conductivity, but also enhance the adsorption of Na_2_S_n_ species and cyclo-S_8_ compared to pristine graphene, which is mainly contributed by the strong chemical bonds of metal-S and N-Na. Among the doping transition metals considered here, Fe-N_4_ co-doped graphene is the best anchoring material. Consequently, the electrocatalytic property of Fe-N_4_@graphene was studied through ci-NEB method, and we found that the doped transition metal iron could dramatically decrease the decomposition energy barrier of Na_2_S (dropped 0.89 eV) and Na_2_S_2_ (dropped 0.18 eV), which has a significantly improvement on the utilization of sulfur and full discharge of batteries. Our findings enrich the fundamental understanding of the micro-level mechanism of metal-N_4_@graphene as multifunctional anchoring materials added to sulfur electrode, and shed light on the design of high-performance Na-S batteries.

## Figures and Tables

**Table 1:** The average bond length (*d*) of isolated Na_2_S_n_ species and cyclo-S_8_. The unit of bond length is angstrom (Å).

	Na_2_S	Na_2_S_2_	Na_2_S_4_	Na_2_S_6_	Na_2_S_8_	Cyclo-S_8_
*d*_Na-S_	2.464	2.581	2.725	2.760	2.734	-
*d*_S-S_	-	2.277	2.135	2.117	2.093	2.097

**Table 2:** The adsorption energies *E*_ad_ (eV) and the average bond length d (Å) of Na_2_S_n_ species and cyclo-S_8_ after absorbing on Fe-N_4_@graphene.

Fe-N_4_@G	Na_2_S	Na_2_S_2_	Na_2_S_4_	Na_2_S_6_	Na_2_S_8_	Cyclo-S_8_
*E*_b_	1.092	1.358	1.072	1.158	1.352	0.924
*d*_Na-N_	2.569	2.790	2.707	2.708	2.920	-
*d*_Fe-S_	-	2.299	2.281	2.299	2.137	2.111
*d*_Na-S_	2.514	2.679	2.747	2.759	2.843	-
*d*_S-S_	-	2.175	2.139	2.064	2.235	2.175

## Data Availability

Data is contained within the article or Supplementary Material.

## Supplementary Materials

The following are available online at https://www.mdpi.com/article/10.3390/nano11051197/s1, Figure S1. Different optimized adsorption configurations of cyclo-S_8_ [(a) and (b)] and Na_2_S_8_ [(c), (d), and (e)] species adsorbed on the surface of Fe-N_4_@graphene. Figure S2. The optimized structures of cyclo-S_8_ and Na_2_S_n_ (n = 1, 2, 4, 6, and 8) species adsorbed on the surface of Co-N_4_@graphene. Figure S3. The optimized structures of cyclo-S_8_ and Na_2_S_n_ (n = 1, 2, 4, 6, and 8) species adsorbed on the surface of Mn-N_4_@graphene. Figure S4. The projected density of states (PDOS) of the doped transition-metal iron. Table S1. The average bond length *d* (Å) and magnetic moment *M* (µ_B_) of transition metal-N_4_@graphene substrates. Table S2. The adsorption energies *E*_ad_ (eV), average bond length *d* (Å), and the minimum distance *d*_min_ (Å) between Na_2_S_n_ species and pristine graphene. Table S3. The adsorption energies *E*_ad_ (eV) and average bond length *d* (Å) of Na_2_S_n_ species and cyclo-S_8_ after adsorbing on Co-N_4_@graphene. Table S4. The adsorption energies *E*_ad_ (eV) and average bond length *d* (Å) of Na_2_S_n_ species and cyclo-S_8_ after adsorbing on Mn-N_4_@graphene.
