# Supplementary material for: Metal-N4@Graphene as Multifunctional Anchoring Materials for Na-S Batteries: First-Principles Study
Source: Nanomaterials (Basel). 2021 May 1;11(5):1197. doi: 10.3390/nano11051197 (PMC8147385; doi:10.3390/nano11051197)
Supplement: Supplementary file 1 [file nanomaterials-11-01197-s001.zip › nanomaterials-1183890-supplementary.pdf]

nanomaterials

Supplementary Materials

Metal-N4@Graphene as Multifunctional Anchoring Materials for Na-S Batteries: First-Principles Study

Kaishuai Yang [1], Dayong Liu [2], Yiling Sun [1,*], Zhengfang Qian [1], Shengkui Zhong [3] and Renheng Wang [1,*]

1 Key Laboratory of Optoelectronic Devices and Systems of Ministry of Education and Guangdong Province, College of Physics and Optoelectronic Engineering, Shenzhen University, Shenzhen 518060, China; ksyang@szu.edu.cn (K.Y.); zq001@szu.edu.cn (Z.Q.)
2 Key Laboratory of Materials Physics, Institute of Solid State Physics, HFIPS, Chinese Academy of Sciences, Hefei 230031, China; dyliu@theory.issp.ac.cn
3 School of Marine Science and Technology, Hainan Tropical Ocean University, Hainan 572000, China; zskui74@163.com
* Correspondence: sunyl@szu.edu.cn (Y.S.); wangrh@szu.edu.cn (R.W.)

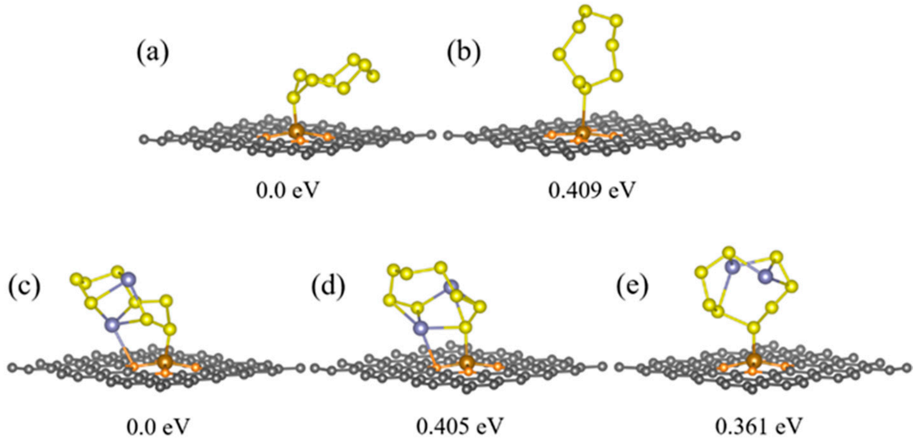

Figure S1. Different optimized adsorption configurations of cyclo-S_8 [(**a**) and (**b**)] and Na_2S_8 [(**c**), (**d**), and (**e**)] species adsorbed on the surface of Fe-N_4@graphene. Their corresponding energies are given below them, and the lowest adsorption energy was set to 0 eV. The black, blue, yellow, orange, and brown balls represent carbon, sodium, sulfur, nitrogen, and iron atoms, respectively.

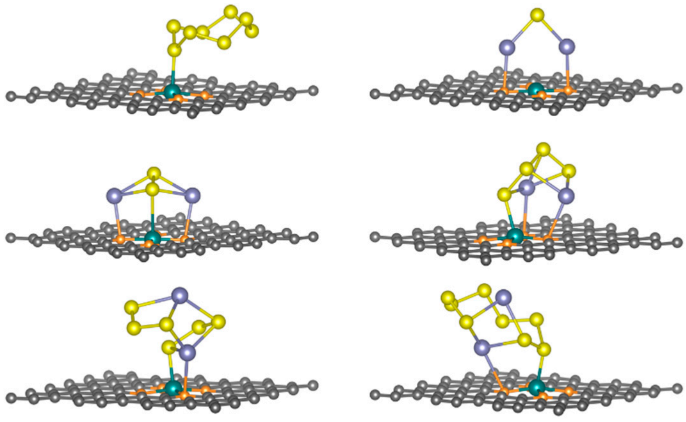

Figure S2. The optimized structures of cyclo-S_8 and Na_2S_n (n = 1, 2, 4, 6, and 8) species adsorbed on the surface of Co-N_4@graphene. The black, blue, yellow, orange, and indigo balls represent carbon, sodium, sulfur, nitrogen, and cobalt atoms, respectively.

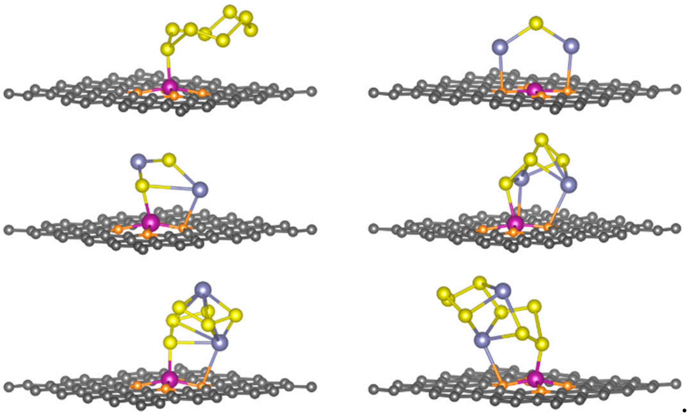

Figure S3. The optimized structures of cyclo-S_8 and Na_2S_n (n = 1, 2, 4, 6, and 8) species adsorbed on the surface of Mn-N_4@graphene. The black, blue, yellow, orange, and magenta balls represent carbon, sodium, sulfur, nitrogen, and manganese atoms, respectively.

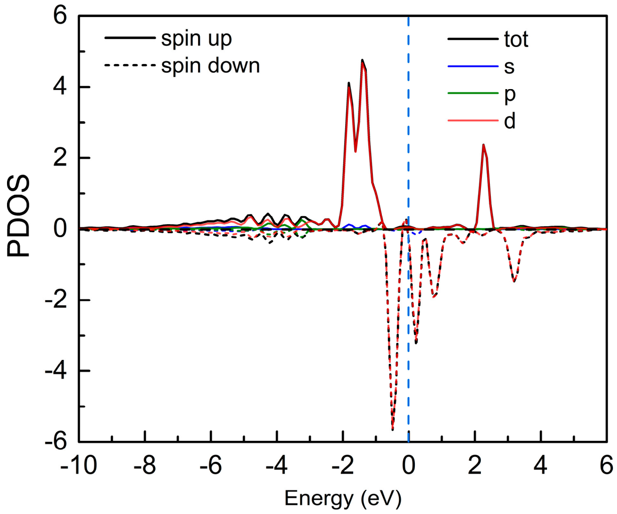

Figure S4. The projected density of states (PDOS) of the doped transition-metal iron. The solid and dashed lines represent spin-up and spin-down component of PDOS, respectively. The vertical blue dashed line represents the Fermi-energy level. The total PDOS near the Fermi-energy level mainly comes from the *d* orbital.

Table S1. The average bond length *d* (Å) and magnetic moment *M* (μ_B) of transition metal-N_4@graphene substrates. The subscript X stands for the C, N, Fe, Co, and Mn atom, respectively.

Metal-N_4@G	C	N	Fe	Co	Mn
$d_{X\text{-}N}$	-	-	1.898	1.894	1.929
$d_{X\text{-}C}$	1.430	1.390	-	-	-
M	0.002	0.030	1.799	1.123	0.638

Table S2. The adsorption energies E_{ad} (eV), average bond length d (Å), and the minimum distance d_{min} (Å) between Na_2S_n species and pristine graphene.

**Graphene**	**Na_2S**	**Na_2S_2**	**Na_2S_4**	**Na_2S_6**	**Na_2S_8**	**cyclo-S_8**
E_b	0.776	1.004	0.626	0.529	0.464	0.742
$d_{Na\text{-}S}$	2.536	2.668	2.745	2.752	2.749	-
$d_{S\text{-}S}$	-	2.156	2.130	2.104	2.131	2.097
d_{min}	2.826	2.871	2.794	3.291	2.850	4.001

Table S3. The adsorption energies E_{ad} (eV) and average bond length d (Å) of Na_2S_n species and cyclo-S_8 after adsorbing on Co-N_4@graphene.

**Co-N_4@G**	**Na_2S**	**Na_2S_2**	**Na_2S_4**	**Na_2S_6**	**Na_2S_8**	**cyclo-S_8**
E_b	0.972	1.127	0.856	1.027	0.924	0.798
$d_{Na\text{-}N}$	2.580	2.820	2.764	2.690	2.906	-
$d_{Co\text{-}S}$	-	2.496	2.484	2.354	2.312	2.472
$d_{Na\text{-}S}$	2.589	2.670	2.738	2.789	2.785	-
$d_{S\text{-}S}$	-	2.166	2.132	2.064	2.170	2.147

Table S4. The adsorption energies E_{ad} (eV) and average bond length d (Å) of Na_2S_n species and cyclo-S_8 after adsorbing on Mn-N_4@graphene.

**Mn-N_4@G**	**Na_2S**	**Na_2S_2**	**Na_2S_4**	**Na_2S_6**	**Na_2S_8**	**cyclo-S_8**
E_b	0.974	1.205	1.026	0.605	0.766	0.837
$d_{Na\text{-}N}$	2.625	2.524	2.769	2.797	2.882	--
$d_{Mn\text{-}S}$	--	2.289	2.456	2.218	2.137	2.252
$d_{Na\text{-}S}$	2.509	2.612	2.702	2.870	2.831	--
$d_{S\text{-}S}$	--	2.204	2.134	2.104	2.178	2.111
